# A multidisciplinary case report of multiple myeloma with renal and cardiac involvement: a look beyond amyloidosis

**DOI:** 10.1186/s12882-022-02984-4

**Published:** 2022-11-17

**Authors:** Samantha Innocenti, Beatrice Bacchi, Marco Allinovi, Federico Perfetto, Elisabetta Antonioli, Niccolo’ Marchionni, Carlo Di Mario, Leonardo Caroti, Francesco Cappelli, Pierluigi Stefàno

**Affiliations:** 1 Nephrology, Dialysis and Transplantation Unit, Careggi University Hospital, Largo Brambilla 3, 50134 Florence, Italy; 2 Department of Cardiac Surgery, Careggi University Hospital, Florence, Italy; 3 Tuscan Regional Amyloid Center, Careggi University Hospital, Florence, Italy; 4 Haematology Unit, Careggi University Hospital, Florence, Italy; 5 Department of Cardiothoracovascular Medicine, Careggi University Hospital, University of Florence, Florence, Italy; 6 Department of Experimental & Clinical Medicine, University of Florence, Florence, Italy; 7 Division of Interventional Structural Cardiology, Cardiothoracovascular Department, Careggi University Hospital, Florence, Italy

**Keywords:** Multiple myeloma, Amyloidosis, Light-chain deposition disease, Renal vein thrombosis, Intracardiac thrombi

## Abstract

**Background:**

Multiple myeloma (MM) is a malignant neoplasm associated with kidney involvement in nearly half of the patients. Cast nephropathy, monoclonal immunoglobulin deposition disease (MIDD), and light chain (AL) amyloidosis are the most common monoclonal immunoglobulin-mediated causes of renal injury.

Cardiac involvement is also present in MM, characterized by restrictive cardiomyopathy generated by light chain deposit or amyloid. Thromboembolic complications such as deep vein thrombosis or pulmonary embolism are also described.

**Case presentation:**

We present an unusual multidisciplinary case of a woman with a newly diagnosed MM associated with severe proteinuria and high natriuretic peptide. A renal and fat pad biopsy with Congo red staining were performed but amyloid deposition was not discovered. While immunofluorescence on fresh frozen unfixed tissue was not contributory, the immunofluorescence on fixed tissue and electron microscopy revealed the correct diagnosis.

During subsequent investigations, two intracardiac right-sided masses and massive pulmonary embolism were also detected.

**Conclusions:**

This case highlights that multiple organ involvement in patients with MM may result from a combination of paraprotein-dependent and -independent factors. Moreover, renal diseases induced by monoclonal gammopathies are a group of complex and heterogeneous disorders. Their subtle presentation and their potential multiorgan involvement require the expertise of a multidisciplinary team able to provide the most appropriate diagnostic and therapeutic assessment.

**Supplementary Information:**

The online version contains supplementary material available at 10.1186/s12882-022-02984-4.

## Background

Multiple myeloma (MM) is a malignant neoplasm associated with kidney involvement in nearly half of the patients [1]. Nephrotic-range proteinuria associated with monoclonal gammopathy can suggest different nephropathies [2]. Differential diagnosis can be extremely difficult due to multiple confounding factors: paraproteins 'masked' on immunofluorescence staining on fresh frozen tissue but positive on paraffin immunofluorescence, coexisting renal vein thrombosis, positive cardiac biomarkers highly suggestive of cardiac amyloidosis, and coexisting clinical aspects frequently associated with secondary focal segmental glomerulosclerosis.

Cardiac involvement is also present in MM, generally characterized by restrictive cardiomyopathy caused by light chain deposit or amyloid (Table 1).

**Table 1:** Cardiac complications in multiple myeloma

**Cardiac complications in multiple myeloma**	
**Amyloid or light chain deposition related:**	**Chemotherapeutic treatment related:**
Restrictive cardiomyopathy	Ischemic heart disease
Pericardial effusion	Congestive heart failure
Pericarditis	Pulmonary hypertension
Cardiac dysfunction	Cardiac dysfunction (irreversibile and dose-related or dose-independent)
Thromboembolism	Thromboembolism
Arrhythmia (atrial fibrillation)	Arrhythmia
Intramiocardial massess	

Here, we describe a patient with a newly diagnosed MM associated with severe proteinuria, atypical cardiac involvement and thromboembolic complications.

## Case presentation

A 52-year-old overweight white woman with hypertension and a 3-years history of chronic kidney disease stage II K-DOQI was referred to our Nephrology department for worsening renal function and resistant hypertension.

Laboratory analysis showed a mild asymptomatic anemia, serum creatinine 1.9 mg/dl, with non-selective nephrotic proteinuria, Bence-Jones proteinuria, and extremely elevated serum kappa free light chains (FLC). Total calcemia was persistently normal and no bone lesions or full-blown nephrotic syndrome were present. Although coagulation profile was persistently not evaluable, probably due to the interfering monoclonal protein, bleeding time was in the normal range (Table 2).

**Table 2:** Patient’s laboratory values

	Normal range	Onset	Month 1	Admission (mo 2)	After surgery	After 1 month of CHT (mo 4)	After 3 months of CHT (mo 6)	After 6 months of CHT (mo 9)	1mo after ASCT (mo 11)
Serum creatinine (mg/dl)	0.50–1.10	1.5	1.9	2.36	1.99	1.31	1.34	1.33	1.28
eGFR (CKD-EPI) (ml/min/1.73mq)	90–140	40	29.8	22.9	28.2	46.7	45.4	46	48
urea (mg/dl)	10–50	N/A	80	110	80	110	N/A	50	50
Hb (g/dl)	12–16	13.2	12.1	13	10.3	11	10.7	12.3	10.5
WBC (10^9/L)	4.0–10.0	8.10	7.24	9.55	12.70	5.60	9.95	5.03	3.25
PLT (10^9/L)	140–440	168	127	118	70	131	117	171	21
INR	0.8–1.2	N/A	N/A	N/A	2.7	2.1	2.6	3.0	2.5
aPTT (sec)	25.0–38.0	N/A	N/A	N/A	25.9	31	N/A	46.1	N/A
Serum immunofixation	Negative	N/A	Kappa FLC	Kappa FLC	Kappa FLC	Kappa FLC	Kappa FLC	Neg	Neg
Kappa light-chain (mg/L)	3.30–19.40	N/A	96 000	93 304	96 256	36.4	17.5	18.76	0.75
Lambda light-chain (mg/L)	5.7–26.30	N/A	5.33	4.64	5.46	21.62	11.98	11.4	1.11
FLC ratio	0.26–1.65	N/A	18 000	21 277	17 629	1.67	1.46	1.64	0.68
D-Dimer (ng/ml)	< 500	N/A	N/A	7541	4796	N/A	436	< 200	< 200
NT-Pro-BNP (pg/mL)	1–125	N/A	455	1242	722	N/A	1 566	N/A	826
Troponin T HS (pg/ml)	< 14	N/A	N/A	328	2298	N/A	28.8	N/A	N/A
Bence-Jones proteinuria (g/24 h)	Negative	N/A	1.71	0.65	1.61	Trace	Neg	Neg	Neg
Proteinuria (g/24 h)	< 0.15	7.0	6.58	2.8	N/A	1.2	3.15	1.07	1.1
β_2_ microglobulin (mg/L)	1.2–2.5	102	133	146	133	5.8	4	N/A	N/A
IL-6 (pg/mL)	0.0–10.0	N/A	N/A	N/A	113.5	74.2	2.4	N/A	N/A
Albumin (g/L)	35–50	N/A	40	35.6	40	27	36.5	45.9	40.0
Total serum protein (g/dL)	6.0–8.2	N/A	N/A	7.6	4.5	5	5.3	6.1	5.8
Total serum calcium (mg/dl)	8.6–10.2	9.3	N/A	9.9	8.8	7.6	9.2	9.6	8.3
IgG (g/L)	7.0–16.0	N/A	N/A	0.91	1.15	2.67	2.15	3.91	3.09
IgA (g/L)	0.7–4.00	N/A	N/A	< 0.07	0.09	0.56	0.55	0.61	< 0.07
IgM (g/L)	0.4–2.30	N/A	N/A	< 0.04	0.05	0.71	0.16	0.23	0.11
Gamma-globulin (%)	11.1–18.8	N/A	N/A	22.2	19.7	5.3	3.8	6.2	6.7
C3 (g/L)	0.90–1.80	N/A	N/A	1.3	N/A	N/A	1.12	N/A	N/A
C4 (g/L)	0.10–0.40	N/A	N/A	0.5	N/A	N/A	0.32	N/A	N/A

*ASCT* autologous stem cell transplantation, *CHT* chemotherapy, *mo* month, *eGFR* estimated Glomerular Filtration Ratio, *FLC* Free light chain, *Hb* Hemoglobin, *IL-6* interleukin 6; INR, International Standardized Ratio, *N/A* not available, *Neg* negative, *PLT* platelet, *WBC* white blood cell

A bone marrow biopsy revealed a complete metaplasia of clonal plasma cells with > 90% of clonal plasma cells and cytogenetic analysis (FISH) confirmed the diagnosis of micromolecular kappa MM with high-risk chromosomal abnormalities, R-ISS 3. All clonal plasma cells carried translocation t(14;16) on IGH/MAF gene. Complete immunoparesis was also noticed, while CRAB criteria were not reported.

Patient showed increased NT-proBNP and high-sensitivity troponin (hs-cTnT) suggesting cardiac involvement. ECG showed tachycardia, first degree AV block and right axial deviation with right conduction delay. Unexpectedly, Congo-red stain on abdominal fat was negative for amyloid deposition. Transthoracic echocardiogram (TTE) revealed a 4.4 × 2.8 cm right atrial mass projecting through the tricuspid valve orifice, and a second 1.5 cm mass located at the right ventricle (RV) apex. Function and motility of both ventricles were preserved, and no sign of left ventricular (LV) hypertrophy was present (interventricular septum 10 mm, LV posterior wall 8 mm). No significant valvular regurgitations were identified.

**Fig. 1:**
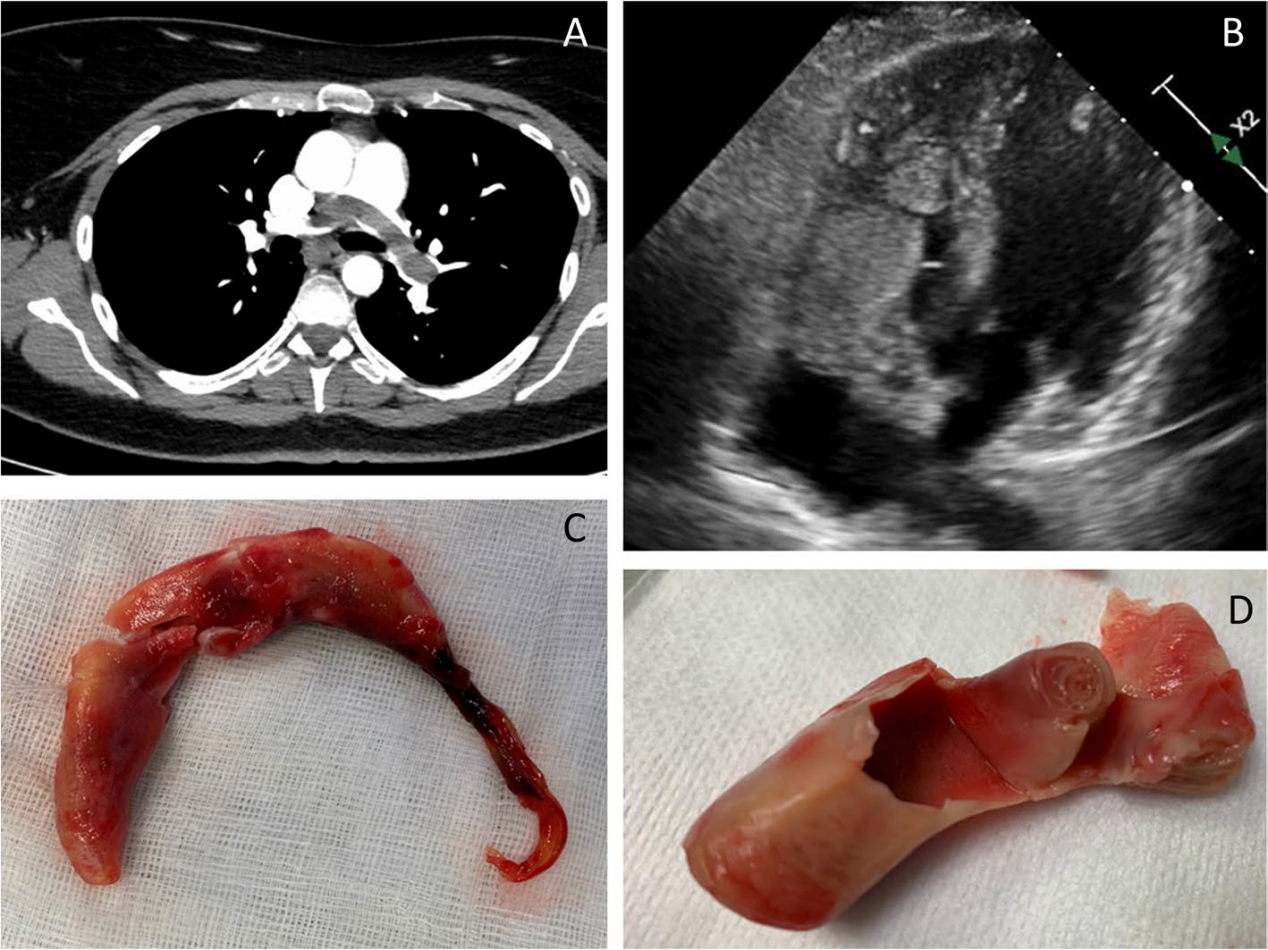
Pulmonary embolism and intracardiac masses. **A**, the thrombus involving the pulmonary trunk; **B**, “*” the right atrial mass projecting through the tricuspid valve orifice, and the mass “#” located at the right ventricle; **C**, **D** pulmonary branch’ and vena cava’ thrombus fragments, respectively

A computed tomography (CT) pulmonary angiogram showed RV thrombi, a large thrombus involving the pulmonary trunk and its two main right and left branches, as well as the segmental basal branches of the left lung. A partial thrombosis was described in the inferior vena cava from its intrahepatic tract to the origin of the renal veins (extended for about 7,5 cm) (Fig. 1). Complete thrombosis of the left renal vein was also detected. Remarkably, the patient reported only mild asthenia, normal blood pressure, no dyspnea, and 99% oxygen saturation in room air. Since she was hemodynamically stable, unfractionated heparin was promptly started but, according to the thrombosis extension and the high risk of embolization, the patient was referred to cardiac surgery.

Through a midline-sternotomy approach, a bilateral pulmonary thrombus was removed en-bloc with attached casts of the lobar branches across an incision in the pulmonary artery.

Both masses from the right chambers were removed through the right atrium (Fig. 1), while the thrombus into the inferior vena cava was too firmly attached to the vessel wall to be extracted. Cardiopulmonary bypass was terminated without inotropic supports. After surgery, patient restarted anticoagulation therapy with unfractionated heparin, subsequently substituted by warfarin.

**Fig. 2:**
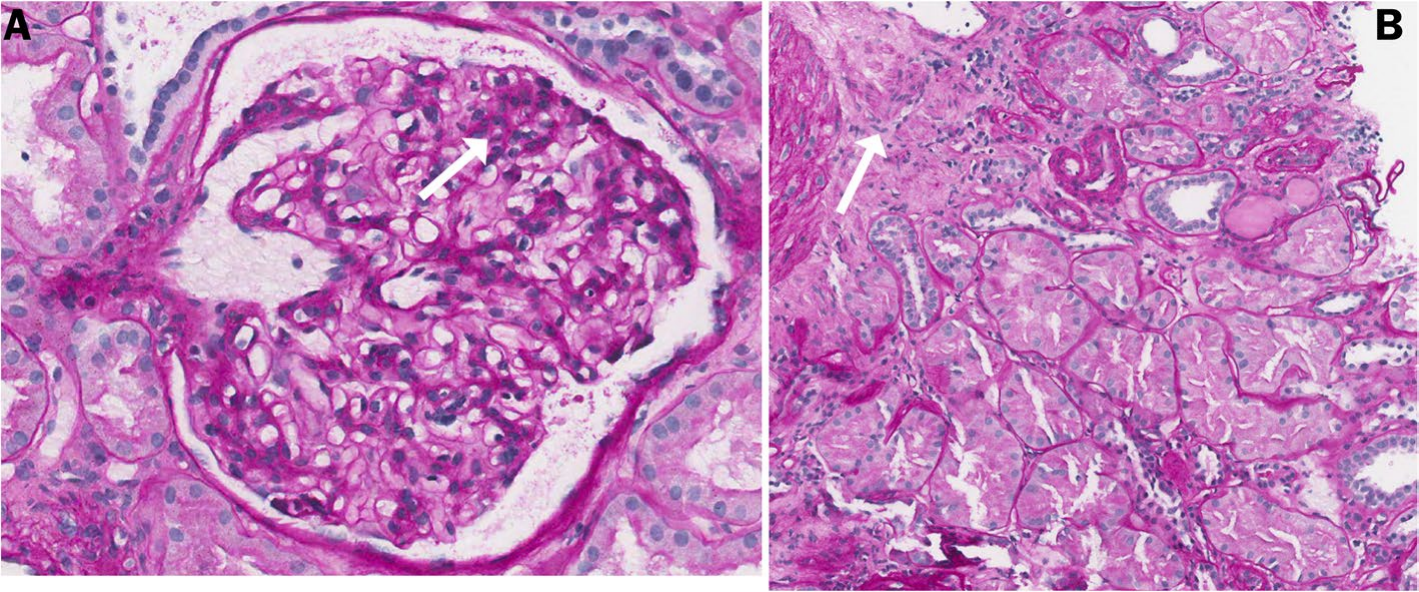
Histopathological findings on light microscopy. **A**: moderately increased glomerular mesangial matrix with mild focal mesangial hypercellularity (white arrow), no significant arteriolar changes, absence of mesangial nodules or nodular glomerulosclerosis (Periodic acid Schiff stain, X400); **B**: small areas of interstitial fibrosis (IF < 25%) and lymphocytic infiltrate (white arrow), aspects of protein reabsorption in renal tubules (Periodic acid Schiff stain, X400)

**Fig. 3:**
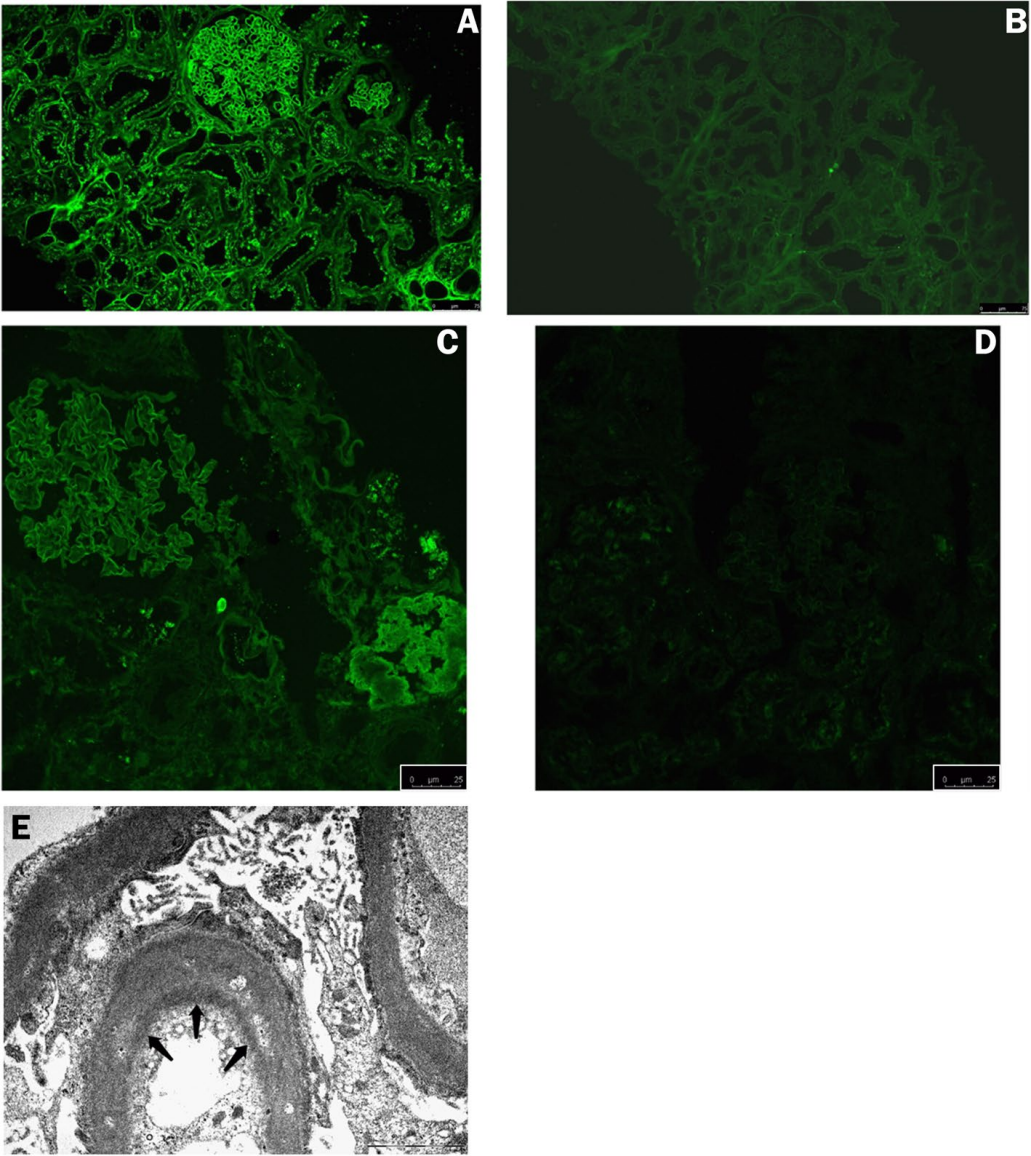
Histopathological findings on immunofluorescence and electron microscopy. Immunofluorescence showed a positive linear tubular and glomerular basement membrane staining (3 +) for kappa (**A**, 20x) and negative for lambda (**B**, 20x) light chains on formalin-fixed paraffin-embedded tissue. Immunofluorescence staining on fresh frozen tissue was weakly positive (± or 1 +) for kappa (**C**, 40x) and negative for lambda (**D**, 40x) light chains. Electron microscopy (**E**, 14000x) showed foot process effacement with cytoplasm vacuolization and ground-pepper-like subendothelial deposits (arrows)

Meantime, a kidney biopsy was performed and light microscopy showed a moderately increased glomerular mesangial matrix without endo or extracapillary proliferation. No morphological lesions such as mesangial nodules or nodular glomerulosclerosis were recognized and none of the glomeruli were sclerotic. There was a grade 1 interstitial fibrosis (IF < 25%) with small areas of lymphocytic infiltrate. Also, rare inflammatory hyaline casts were found in the tubules in the absence of concurrent cast-nephropathy. Vascular compartment was practically normal according to patient’s age (Fig. 2). Congo red staining was once again negative. Immunofluorescence (IF) on fresh frozen unfixed tissue was not contributory, with only weak (± or 1 +) staining for C3 and kappa FLC (Fig. 3C, D). Differently, IF on fixed tissue demonstrated an intense (3 +) linear staining for kappa FLC along the glomerular and tubular basement membranes, while IgG, lambda, and C3 staining were negative (Fig. 3A, B). Electron microscopy showed segmentary “ground pepper-like” deposits in the subendothelial space and the glomerular basement membranes (GBM). Similar deposits were observed along the tubular basement membrane (TBM). Extensive podocyte foot process effacement was seen with no sub-epithelial or mesangial electron-dense deposits (Fig. 3E). The final diagnosis was “kappa light chain deposition disease (LCDD)”.

The patient fully recovered from surgery. A new TTE showed preserved function of both ventricles (EF 58%, TAPSE 20 mm, RV-RA gradient 25 mmHg) or major valvular disease. No new intracardiac masses were detected (video, Additional file 1).

A 3-months follow-up CT showed the persistence of only a partially calcified thrombus in the right pulmonary artery’s distal branches, warfarin was continued.

After 4 cycles of VTD protocol (Bortezomib, Thalidomide, Dexamethasone), the patient presented a very-good partial hematologic remission. Afterwards, she received autologous hematopoietic stem cell transplantation, with a stable complete hematologic remission and a progressive improvement of proteinuria and renal function (Table 2).

## Discussion and conclusions

This case proves that a step-by-step diagnostic flow chart and a multidisciplinary clinical evaluation are crucial to obtain the right diagnosis.

At the time of admission, the worsening of renal function with nephrotic-range proteinuria, elevated kappa FLC, increase NT-proBNP and hs-cTnT strongly suggested AL systemic amyloidosis with both renal and cardiac involvement. However, Congo red staining negativity of two biopsies, made a mandatory reassessment of differential diagnosis for cardiac and renal involvement.

Nephrotic range proteinuria without the full-blown nephrotic syndrome could suggest secondary/maladaptive focal segmental glomerulosclerosis, in particular when one or more risk factors are present, such as for obesity and reduced renal parenchymal mass [3], as observed in our patient. Moreover, the left renal vein thrombosis, observed on CT, could have explained at least in part the degree of proteinuria [4].

In the context of monoclonal gammopathies of renal significance, not all patients with high levels of paraprotein present with reduced renal function, although FLC levels > 800 mg/L are good predictors of severe renal failure [5]. However, despite the extremely high levels of kappa FLC, our patient showed only a mild-to-moderate worsening of kidney function and no histological signs of cast nephropathy. In fact, physicochemical properties of the secreted paraprotein may determine pathological features, for which a variety of Ig-dependent and -independent mechanisms have been described [6].

Among patients with monoclonal gammopathies, those presenting with heavy proteinuria and milder renal impairment are more likely to have AL amyloidosis, LCDD or HCDD [7]. Excluding the first, patients with LCDD usually present with proteinuria (nephrotic-range proteinuria is seen in about 50% of cases), microscopic hematuria, hypertension, and variable degrees of renal insufficiency. Clinical presentation depends on several histopathological aspects: the site of the FLC deposition in renal compartments, the extent of chronic lesions, the degree of foot process effacement, and overlap with myeloma cast nephropathy [2].

The IF is essential for the definitive diagnosis of LCDD. However, there are rare cases (as in our patient) in which the immune deposits and paraproteins are 'masked' on routine IF, resulting in false-negative staining on fresh frozen tissue, and paraffin immunofluorescence can be used to unmask FLC deposits [8]. LCDD diagnosis via kidney biopsy permitted to establish an early and correct chemotherapy regimen that led to a complete hematologic response, which is mandatory to improve renal and global outcomes.

In patients with clinical suspicion of AL amyloidosis or LCDD, increased NT-proBNP and hs-cTnT represent sensitive markers to identify cardiac involvement [9]. Surprisingly, echocardiography showed no signs of cardiac dysfunction [10], in particular no increased wall thickness, or diastolic dysfunction while, it demonstrated multiple right-sided cardiac masses. According to the patient’s history and masses aspects, only a few hypotheses were acceptable: heart thrombi [11], mobilized deep venous thrombi, and, less likely, primary or metastatic tumors [12].

In our case, since both right chambers were involved, a metastasis from a primary neoplasm (renal-cell carcinoma or hepatocellular carcinoma) extended through the inferior vena cava to the right side of the heart should be also considered. However, no evidence of renal or hepatic lesions was appreciated on an abdominal CT.

Of note, the right atrium is probably the predominant location of plasmacytoma involving the heart but it is a rare presentation of MM [13].

In our patient, histological examination of the intracardiac masses confirmed the thrombotic nature.

Among different complications of MM a high risk of venous thrombosis has been previously described. The thrombophilic state is multifactorial and often divided in three categories: (i) malignancy-related: is potentially characterized by the hyperviscosity syndrome due to increased paraprotein content, the release of inflammatory cytokines (as IL-6), and several changes in coagulation (as an increased von Willebrand factor or factor VIII) [14]; (ii) patient-related: such as the presence of central venous access devices, hypoalbuminemia, renal failure, immobilization and obesity [15], and (iii) therapy-related: as during treatment with immunomodulatory drugs (thalidomide lenalidomide and pomalidomide) which have a prothrombotic effect. Current literature lacks of data about a possible direct pathogenetic role of paraproteins in venous thrombosis [16]. In some case reports, the monoclonal light chain is identified as an interfering factor in functional assays and coagulation tests causing dysfibrinogenemia [17]. In our case, a lot of contributory factors are involved in the development of the prothrombotic state, such as obesity, very high levels of free light chains and hypoalbuminemia.

Considering the extension of the thrombosis and the plausible chronic state, anticoagulant therapy alone was considered insufficient.

In case of acute pulmonary embolism with hemodynamic instability, thrombolysis is recommended while surgical embolectomy is considered as an alternative in patients not responsive to thrombolytic therapy or with acute hemodynamic deterioration. Surgical thrombosis removal, instead, is the treatment of choice in chronic thrombosis of the pulmonary tree [18]. In our report, the operability of the patient was approved by a multidisciplinary team after evaluation of several parameters: NYHA class, the risk of rapid hemodynamic deterioration, and the patient’s *quoad vitam* prognosis. Therefore, surgical thrombectomy was considered the best option. Moreover, the heart surgical intervention was crucial in order to prevent acute RV dysfunction, recurrent pulmonary embolism and thus cardiogenic shock.

The natural history and prognosis of MIDD depend on the severity of renal failure at diagnosis, the presence of an underlying MM, and the delay in the hematologic response to chemotherapy. Additionally, LCDD patients with cardiac involvement have poorer survival and a significantly higher risk of treatment-related mortality after ASCT [19]. Moreover, our patient showed several parameters associated with unfavorable MM outcome. Some negative prognostic factors are widely accepted, such as high-risk chromosomal abnormalities, high serum β2-microglobulin (≥ 5.5 mg/L), and low serum albumin [20]. Other prognostic factors are not widely validated, such as immunoparesis, which have a negative impact on the progression-free survival [21], high serum IL-6 levels [22], or extremely high levels of FLC [23], which have been shown to play a prominent role in the development of kidney damage.

Overall, both early diagnosis and prompt treatment with bortezomib and ASCT-based combinations can improve the prognosis of LCDD, by reducing circulating immunoglobulins, preserving renal function, and improving overall survival, even in patients with a severe disease at onset.

In conclusion, in patients with MM, multiple organ involvement may result from a combination of paraprotein-dependent and -independent factors, and the therapeutic success requires the early recognition of all the pathogenetic elements involved. This case reminds that sometimes, to reach the right diagnosis, looking beyond the surface is mandatory. Moreover, in patients with not acute massive pulmonary embolism and intracardiac right masses, surgical pulmonary embolectomy should be promptly performed to preserve RV function and prevent pulmonary hypertension development. This case also demonstrated that both early diagnosis and prompt treatment with bortezomib and ASCT-based combinations can improve the prognosis of LCDD, even in patients with a severe disease at onset.

## Supplementary Information

**Additional file 1.** Intracardiac thrombi. The video shows echocardiography performed before and after surgery.  

## Data Availability

The datasets used and/or analysed during the current study are available from the corresponding author on reasonable request.

## Abbreviations

MM: Multiple myeloma
FLC: Free light chains
TTE: Transthoracic echocardiogram
RV: Right ventricle
LV: Left ventricle
CT: Computed tomography
IF: Immunofluorescence
LCDD: Light chain deposition disease

## References

[1] Joly F, Cohen C, Javaugue V, Bender S, Belmouaz M, Arnulf B, Knebelmann B, Nouvier M, Audard V, Provot F, Gnemmi V, Nochy D, Goujon JM, Jaccard A, Touchard G, Fermand JP, Sirac C, Bridoux F (2019). Randall-type monoclonal immunoglobulin deposition disease: novel insights from a nationwide cohort study. Blood.

[2] Lin J, Markowitz GS, Valeri AM, Kambham N, Sherman WH, Appel GB, D'Agati VD (2001). Renal monoclonal immunoglobulin deposition disease: the disease spectrum. J Am Soc Nephrol.

[3] Kopp JB, Anders HJ, Susztak K, Podestà MA, Remuzzi G, Hildebrandt F, Romagnani P (2020). Podocytopathies. Nat Rev Dis Primers.

[4] Morrissey EC, McDonald BR, Rabetoy GM (1997). Resolution of proteinuria secondary to bilateral renal vein thrombosis after treatment with systemic thrombolytic therapy. Am J Kidney Dis.

[5] Yadav P, Cockwell P, Cook M, Pinney J, Giles H, Aung YS, Cairns D, Owen RG, Davies FE, Jackson GH, Child JA, Morgan GJ, Drayson MT (2018). Serum free light chain levels and renal function at diagnosis in patients with multiple myeloma. BMC Nephrol.

[6] Heher EC, Rennke HG, Laubach JP, Richardson PG (2013). Kidney disease and multiple myeloma. Clin J Am Soc Nephrol.

[7] Montseny JJ, Kleinknecht D, Meyrier A, Vanhille P, Simon P, Pruna A, Eladari D (1998). Long-term outcome according to renal histological lesions in 118 patients with monoclonal gammopathies. Nephrol Dial Transplant.

[8] Nasr SH, Fidler ME, Said SM (2018). Paraffin immunofluorescence: a valuable ancillary technique in renal pathology. Kidney Int Rep.

[9] Palladini G, Barassi A, Klersy C, Pacciolla R, Milani P, Sarais G, Perlini S, Albertini R, Russo P, Foli A, Bragotti LZ, Obici L, Moratti R, Melzid'Eril GV, Merlini G (2010). The combination of high-sensitivity cardiac troponin T (hs-cTnT) at presentation and changes in N-terminal natriuretic peptide type B (NT-proBNP) after chemotherapy best predict survival in AL amyloidosis. Blood.

[10] Buxbaum JN, Genega EM, Lazowski P, Kumar A, Tunick PA, Kronzon I, Gallo GR (2000). Infiltrative nonamyloidotic monoclonal immunoglobulin light chain cardiomyopathy: an underappreciated manifestation of plasma cell dyscrasias. Cardiology.

[11] Martinez-Naharro A, Gonzalez-Lopez E, Corovic A, Mirelis JG, Baksi AJ, Moon JC, Garcia-Pavia P, Gillmore JD, Hawkins PN, Fontana M (2019). High Prevalence of intracardiac thrombi in cardiac amyloidosis. J Am Coll Cardiol.

[12] Poterucha TJ, Kochav J, O’Connor DS, Rosner GF (2019). Cardiac tumors: clinical presentation, diagnosis, and management. Curr Treat Options Oncol..

[13] Fernandez LA, Couban S, Sy R, Miller R (2001). An unusual presentation of extramedullary plasmacytoma occurring sequentially in the testis, subcutaneous tissue, and heart. Am J Hematol.

[14] Kwaan HC (2013). Hyperviscosity in plasma cell dyscrasias. Clin Hemorheol Microcirc.

[15] Leebeek FW (2016). Update of thrombosis in multiple myeloma. Thromb Res.

[16] Auwerda JJ, Sonneveld P, de Maat MP, Leebeek FW (2007). Prothrombotic coagulation abnormalities in patients with paraprotein-producing B-cell disorders. Clin Lymphoma Myeloma.

[17] Martini F, Cecconi N, Paolicchi A (2019). Interference of monoclonal gammopathy with fibrinogen assay producing spurious dysfibrinogenemia. TH Open.

[18] Konstantinides SV, Meyer G, Becattini C, Bueno H, Geersing GJ, Harjola VP, Huisman MV, Humbert M, Jennings CS, Jiménez D, Kucher N, Lang IM, Lankeit M, Lorusso R, Mazzolai L, Meneveau N, NíÁinle F, Prandoni P, Pruszczyk P, Righini M, Torbicki A, Belle V, ESC Scientific Document Group (2020). 2019 ESC Guidelines for the diagnosis and management of acute pulmonary embolism developed in collaboration with the European Respiratory Society (ERS). Eur Heart J.

[19] Mohan M, Buros A, Mathur P, Gokden N, Singh M, Susanibar S, Jo Kamimoto J, Hoque S, Radhakrishnan M, Matin A, Davis C, Grazziutti M, Thanendrarajan S, van Rhee F, Zangari M, Davies F, Morgan G, Epstein J, Barlogie B, Schinke C (2017). Clinical characteristics and prognostic factors in multiple myeloma patients with light chain deposition disease. Am J Hematol.

[20] Lonial S, Boise LH, Kaufman J (2015). How I treat high-risk myeloma. Blood.

[21] Heaney JLJ, Campbell JP, Iqbal G, Cairns D, Richter A, Child JA, Gregory W, Jackson G, Kaiser M, Owen R, Davies F, Morgan G, Dunn J, Drayson MT (2018). Characterisation of immunoparesis in newly diagnosed myeloma and its impact on progression-free and overall survival in both old and recent myeloma trials. Leukemia.

[22] Pelliniemi TT, Irjala K, Mattila K, Pulkki K, Rajamäki A, Tienhaara A, Laakso M, Lahtinen R (1995). Immunoreactive interleukin-6 and acute phase proteins as prognostic factors in multiple myeloma. Finnish Leukemia Group. Blood.

[23] Chilkulwar A, Mewawalla P, Miller A, Berteotti G, Sahovic E, Lister J (2016). Serum free light chain concentration (>1000 mg/dl) at diagnosis and at relapse predicts for very poor prognosis in multiple myeloma. Blood.

